# Longitudinal significance of six-minute walk test in patients with nontuberculous mycobacterial pulmonary disease: an observational study

**DOI:** 10.1186/s12890-023-02528-y

**Published:** 2023-07-06

**Authors:** Atsuho Morita, Kazuma Yagi, Takanori Asakura, Ho Namkoong, Yasunori Sato, Takunori Ogawa, Tatsuya Kusumoto, Shoji Suzuki, Hiromu Tanaka, Ho Lee, Satoshi Okamori, Shuhei Azekawa, Kensuke Nakagawara, Masanori Kaji, Genta Nagao, Yohei Funatsu, Yoshifumi Kimizuka, Hirofumi Kamata, Tomoyasu Nishimura, Makoto Ishii, Koichi Fukunaga, Naoki Hasegawa

**Affiliations:** 1 Division of Pulmonary Medicine, Department of Medicine, Keio University School of Medicine, Tokyo, Japan; 2 Department of Clinical Medicine (Laboratory of Bioregulatory Medicine), Kitasato University School of Pharmacy, Tokyo, Japan; 3 Department of Respiratory Medicine, Kitasato University, Kitasato Institute Hospital, Tokyo, Japan; 4 Department of Infectious Diseases, Keio University School of Medicine, Tokyo, Japan; 5 Department of Preventive Medicine and Public Health, Keio University of Medicine, Tokyo, Japan; 6 Department of Internal Medicine, Tachikawa Hospital, Tokyo, Japan; 7 Division of Infectious Diseases and Respiratory Medicine, Department of Internal Medicine, National Defense Medical College, Saitama, Japan; 8 Keio University Health Center, Tokyo, Japan; 9 Department of Respiratory Medicine, Nagoya University Graduate School of Medicine, Nagoya, Japan

**Keywords:** Nontuberculous mycobacteria, Nontuberculous mycobacterial pulmonary disease, Six-minute walk test, Six-minute walk distance, Health-related quality of life, St. George’s respiratory questionnaire

## Abstract

**Background:**

The long-term exercise tolerance changes in patients with nontuberculous mycobacterial pulmonary disease (NTM-PD) are of great interest because of its chronic course. This study aimed to characterize the associations between changes over time in six-minute walking test (6MWT) parameters and clinical parameters in patients with NTM-PD.

**Methods:**

Overall, 188 patients with NTM-PD, visiting outpatient clinics at Keio University Hospital from April 2012 to March 2020 were included in the study. Data were collected using the St. George’s Respiratory Questionnaire (SGRQ), pulmonary function test (PFT), blood tests, and the 6MWT at registration and at least once after that. The association of the anchors and clinical indicators with the 6MWT parameters was assessed.

**Results:**

The median age [interquartile range] of the patients was 67 [63–74] years. The median baseline six-minute walk distance (6MWD) and final Borg scale (FBS) were 413 [361–470] m and 1 [0–2], respectively. In the correlation analysis, ΔSGRQ total/year (yr), Δforced vital capacity (FVC, % predicted)/yr, Δforced expiratory volume in 1 s (FEV_1_, % predicted)/yr, and Δdiffusing capacity for carbon monoxide (DL_CO_, % predicted)/yr correlated with both Δ6MWD/yr and ΔFBS/yr in the longitudinal analysis (|*Rho*| > 0.20). When stratified into three quantiles of changes in each anchor, the 6MWT parameters worsened over time in the bottom 25% group by mixed-effects model. Specifically, Δ6MWD was affected by SGRQ activity, SGRQ impacts, PFT (FVC, FEV_1_, and DL_CO_), and C-reactive protein (CRP). ΔFBS was affected by all SGRQ components, total score, and PFT. Anchor scores and variables at baseline that worsened Δ6MWD were higher SGRQ scores, lower FVC (% predicted), lower DL_CO_ (% predicted), higher Krebs von den Lungen-6, old age, and undergoing treatment at registration. Similarly, these clinical parameters and elevated CRP, excluding undergoing treatment at registration, worsened ΔFBS.

**Conclusions:**

The decreased walking distance and exacerbation of dyspnea on exertion over time in patients with NTM-PD may reflect a deterioration of health-related quality of life and pulmonary function. Thus, the change in 6MWT over time can be used as an indicator to accurately assess the patient’s condition and tailor their healthcare environment.

**Supplementary Information:**

The online version contains supplementary material available at 10.1186/s12890-023-02528-y.

## Background

The prevalence of nontuberculous mycobacterial pulmonary disease (NTM-PD) is increasing worldwide [1, 2]. NTM-PD, the most common form of NTM infection, usually presents as a chronic and slowly progressing disease in immunocompetent patients [3, 4]. NTM-PD is generally incurable, requires long-term antimicrobial treatment, and has a high recurrence rate after treatment discontinuation [3–5]; therefore, patients with NTM-PD suffer from its symptoms, such as bloody sputum and dyspnea, as well as associated limitations in activities of daily living (ADL), impact on social activities, and psychological burden [6, 7]. Because of the increasing chronicity of NTM-PD, monitoring the overall health status of affected patients using patient-reported outcome measures that represent the health-related quality of life (HRQL) has become clinically important [8]. Our group previously reported that HRQL, particularly the physical component, is impaired in patients with NTM (*Mycobacterium avium* complex [MAC])-PD [6]. Furthermore, we recently revealed that HRQL evaluated using St. George’s Respiratory Questionnaire (SGRQ) showed longitudinal validity in assessing disease activity and was associated with changes in the pulmonary function test (PFT) parameters in patients with MAC-PD [9].

The six-minute walk test (6MWT) is a standardized exercise test for the assessment of cardiopulmonary diseases because of its simplicity, low cost, non-invasiveness, ease of use, and reproducibility [10, 11]. Therefore, it has become a useful and objective scale for assessing exercise capacity in daily life and predicting the prognosis of patients with chronic pulmonary diseases [12–15]. Moreover, previous studies including patients with NTM-PD have demonstrated associations between six-minute walk distance (6MWD) and HRQL parameters evaluated using SGRQ [16, 17]. The final Borg scale (FBS) at the end of 6MWT is also useful in assessing different aspects of dyspnea [18]. Furthermore, each point of the modified Borg scale is equidistant and is excellent for detecting changes over time for the same patient [19]. However, not only have there been no studies of long-term recording and analysis of 6MWT parameters in NTM-PD patients, but also the meaning of the changes over time is unknown. Therefore, this study aimed to characterize the associations between changes over time in 6MWT parameters and clinical parameters, including HRQL (SGRQ), PFT, and blood test (BT) findings in patients with NTM-PD.

## Methods

### Study patients and design

We conducted a prospective observational study at Keio University Hospital (UMIN000007546) [9, 20–24] from April 2012 to March 2020 that included outpatients aged ≥ 20 years with diagnosed or suspected NTM-PD based on the American Thoracic Society (ATS)/Infectious Disease Society of America (IDSA) statements published in 2007 [3]. This study protocol was approved by the Ethics Review Board of Keio University Hospital (No. 20,110,267), and all patients provided written informed consent.

Clinical parameters including sex, age at diagnosis, disease duration, body mass index (BMI), smoking status, underlying pulmonary diseases, pathogens, and comorbidities assessed using the age-adjusted Charlson comorbidity index [25], sputum smear and culture findings for NTM within the previous year, and treatment status were recorded at registration. NTM isolates from sputum were identified as described previously [22, 24]. Patients underwent blood tests, PFTs, high-resolution computed tomography (HRCT) examinations, SGRQ assessment of HRQL, and 6MWT at registration and subsequently once a year.　Baseline was defined as the first year wherein data for both 6MWT and SGRQ were available. All patients were observed until the end of this study (March 2020), the date of their last visit, or their death.

### Measurements

We selected anchors distributed across SGRQ domains (symptom, activity, impact, and total), PFT results (forced vital capacity [FVC], forced expiratory volume in 1 s [FEV_1_], and diffusing capacity for carbon monoxide [DL_CO_]), and BT findings (serum C-reactive protein [CRP], and sialylated carbohydrate antigen Krebs von den Lungen-6 [KL-6]). Patients with NTM-PD had impaired SGRQ parameters with longitudinal validity in terms of disease activity and sensitivity to changes in %FEV_1_ [9]. Previous studies have also reported that FVC and FEV_1_ deteriorate in patients with NTM-PD [26, 27], and DL_CO_ is associated with the severity of pulmonary involvement [28]. CRP has been reported to be a factor associated with SGRQ parameters [6], while KL-6 is considered associated with disease progression and treatment response in patients with NTM-PD [20].

The SGRQ is a self-administered, respiratory-specific questionnaire and has been validated in patients with NTM-PD [6, 17]. It contains 50 items distributed across symptom, activity, and impact domains, and the total score [29]; the total score and the scores in these three domains were calculated. The scores range from 0 to 100 and lower scores indicate a better health status. PFTs were performed for stable patients using an electronic spirometer (Chestac-9800 or HI-801; Chest M.I., Tokyo, Japan). For the 6MWT measurement, patients were instructed to walk in a hallway for 6 min according to the ATS guidelines, and the 6MWD and FBS (as a dyspnea scale) were recorded [10, 19]. To track changes over time for each patient, we evaluated the 6MWT parameters at baseline and the difference in 6MWD and FBS from baseline to the time when the last 6MWT was performed (Δ6MWD, ΔFBS). Finally, patients were stratified into three quantiles: the top 25% (Q1), middle 50% (Q2), and bottom 25% (Q3) groups based on the annual change of anchor (Δanchor/yr).

### Statistical analysis

Descriptive variables were summarized as median, interquartile ranges for continuous variables, and frequency and proportions for categorical variables. To assess the correlation between the 6MWT parameters and each anchor variable cross-sectionally and longitudinally, the values at baseline and the value changes (Δ) from baseline to the time of the last 6MWT measurement were calculated using Spearman correlation coefficients. We further used a mixed-effects model with patients as a random effect for assessing the within-patient correlation of repeated measures over time. We calculated standard errors and confidence intervals using robust estimation methods. After examining the effect of baseline values of anchors and clinical variables, we estimated the mean Δ6MWD or ΔFBS at each time point among the three groups based on the annual change in anchor from baseline to final implementation. The Kenward–Roger approximation was used to estimate denominator degrees of freedom. The linear mixed-effects model is the most widely used method for analyzing longitudinal data with complications of incomplete measurements in a natural way [30]. All *P*-values were two-tailed, and *P* < 0.05 was considered statistically significant. All statistical analyses were performed using JMP version 15.0 (SAS Institute, Cary, NC, USA).

## Results

### Baseline characteristics

**Fig. 1:**
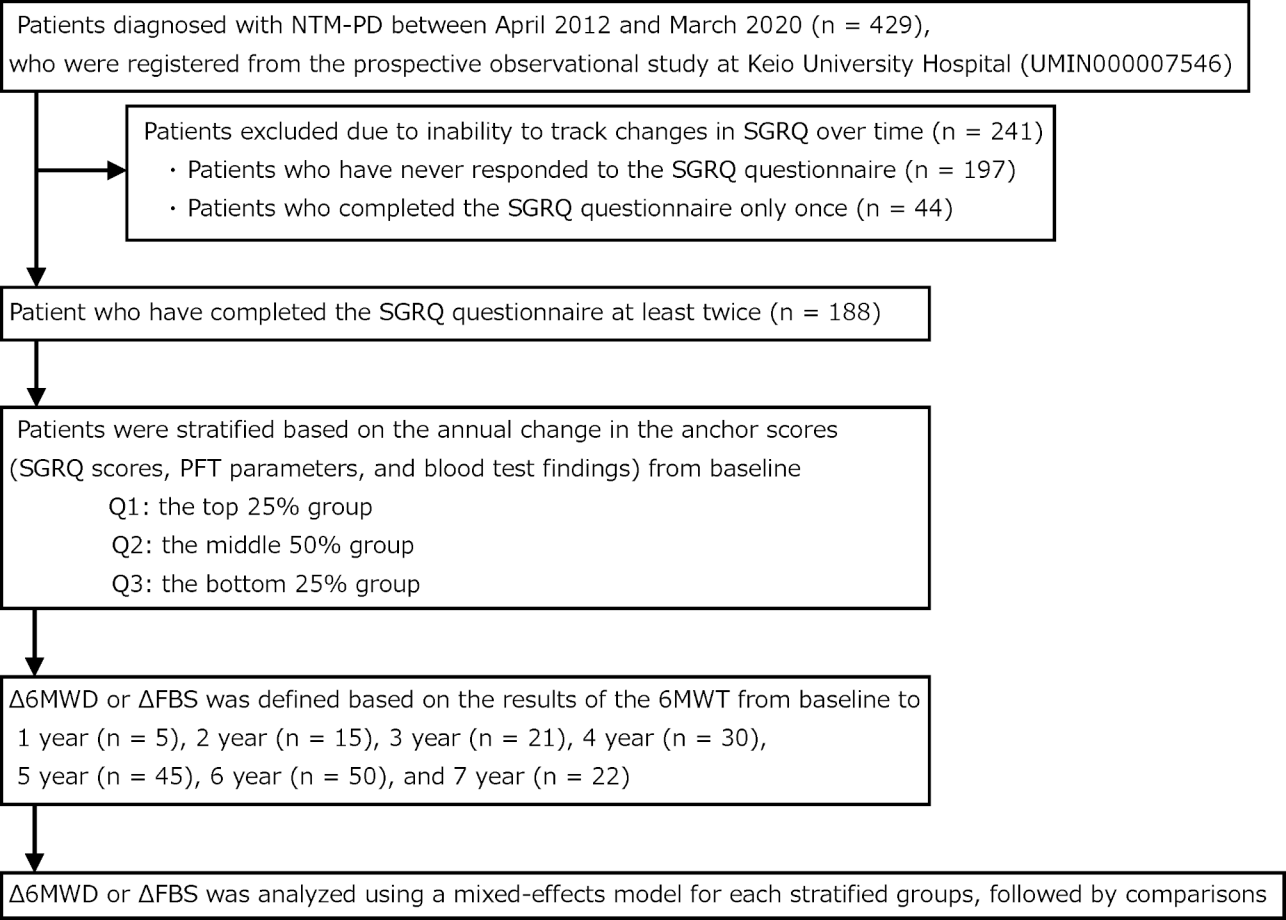
Patient enrollment process for this study. FBS, final Borg scale; NTM, nontuberculous mycobacteria; PD, pulmonary disease; SGRQ, St. George’s Respiratory Questionnaire; PFT, pulmonary function test; 6MWD, 6-minute walk distance; 6MWT, 6-minute walk test

Figure 1 depicts the patient enrollment process of this study. Of the 429 patients with NTM-PD, we excluded 241 patients without complete SGRQ (n = 197) and those who completed the questionnaire only once (n = 44). Finally, we included 188 patients with NTM-PD with at least 2 6MWT and SGRQ measurements to evaluate the association between 6MWT and SGRQ or clinical parameters.

The clinical characteristics of the patients are presented in Table 1. The median (interquartile range [IQR]) age of patients was 67 [63–74] years, and 157 (84%) patients were women. Of these, 167 (89%) were never smokers and 21 (11%) had underlying pulmonary diseases. The most common NTM pathogen in this study was MAC (94%). Positive sputum smear and culture results for NTM within the previous year were noted in 58 (31%) and 103 (55%) patients, respectively. The PFT results were within the normal range except for %DL_CO_. In HRCT performed at registration, the NB pattern was the most observed radiographic pattern (89%); 34 (18%) patients had cavitary lesions. The median [IQR] baseline 6MWD was 413 [361–470] m. The median baseline SGRQ symptom, activity, impact, and total scores were 27.7 [17.1–45.1], 23.3 [6.2–41.5], 8.1 [1.7–24.4], and 15.2 [8.2–32.7], respectively.

**Table 1:** Baseline characteristics of patients with nontuberculous mycobacterial pulmonary disease (n = 188)

Variables and anchors	Patients (n = 188)
Age, years	67 [63–74]
Sex, Male / Female	31 (16) / 157 (84)
Disease duration^*^, years	6.8 [3.7–10.4]
BMI, kg/m^2^	19.4 [17.7–21.0]
Smoking status	
Never/ Former or Currently	167 (89) / 21 (11)
Age-adjusted Charlson comorbidity index	3 [3, 4]
Malignancy	28 (15)
Diabetes mellitus	12 (6)
Underlying pulmonary diseases Old pulmonary tuberculosis Bronchial asthma COPD Lung cancer	21 (11) 9 (5) 5 (3) 1 (1) 3 (2)
Pathogens MAC *M. kansasii* *M. abscessus* complex *M. fortuitum* *M. gordonae* *M. lentiflavum* *M. scrofulaceum* Unidentified	176 (94) 0 (0) 3 (2) 2 (1) 2 (1) 2 (1) 1 (1) 2 (1)
Sputum findings for NTM infection within the previous year^†^	
Smear / Culture positivity	58 (32) / 103 (56)
Treatment status	
Never/ Previously/ Currently	77 (41) / 43 (23) / 68 (36)
FVC^‡^, L	2.55 [2.13–2.93]
FVC^‡^, % predicted	94.5 [82.9–103.8]
FEV_1_^‡^, L	1.79 [1.52–2.24]
FEV_1_^‡^, % predicted	91.2 [78.7–101.9]
DL_CO_^‡^, mL/min/mmHg	14.2 [11.7–16.3]
DL_CO_^‡^, % predicted	67.9 [57.2–77.1]
CRP^¶^, mg/dl	0.1 [0.0–0.2]
KL-6^†^, U/ml	258 [209–361]
Presence of cavitary lesions	34 (18)
Radiological pattern NB/ FC/ NB + FC/ unclassified	167 (89) / 3 (2) / 7 (4) / 11 (6)
6MWD, m	413 [361–470]
Final Borg Scale	1 [0–2]
SGRQ symptom SGRQ activity SGRQ impact SGRQ total	27.7 [17.1–45.1] 23.3 [6.2–41.5] 8.1 [1.7–24.4] 15.2 [8.2–32.7]

BMI, body mass index; COPD, chronic obstructive pulmonary disease; CRP, serum C-reactive protein; DL_CO_, diffusing capacity of the lung for carbon monoxide; FC, fibrocavitary; FEV_1_, forced expiratory volume in 1s; FVC, forced vital capacity; KL-6, sialylated carbohydrate antigen Krebs von den Lungen-6; MAC, *Mycobacterium avium* complex; NB, nodular bronchiectatic; NTM, non-tuberculous mycobacteria; SGRQ, St. George’s Respiratory Questionnaire; 6MWD, 6-minute walk distance. Data are shown as number (%) of patients or medians [interquartile ranges] ^*^From diagnosis to registration;^†^n = 184; ^‡^n = 185; ^§^n = 183; ^||^n = 142; ^¶^n = 186.

### Cross-sectional and longitudinal correlation between anchors and the 6MWD

Table 2 and S1 show the longitudinal and cross-sectional association between the anchor values and the 6MWD, respectively. In the longitudinal analysis, ΔSGRQ activity/year (yr) and ΔSGRQ total/yr were significantly and inversely correlated with Δ6MWD/yr (‘activity’ *Rho* = − 0.30; ‘total’ *Rho* = − 0.22). ΔFVC (% predicted)/yr, ΔFEV_1_ (% predicted)/yr, and ΔDL_CO_ (% predicted)/yr were positively correlated with Δ6MWD/yr (‘Δ%FVC/yr’ *Rho* = 0.29; ‘Δ%FEV_1_/yr’ *Rho* = 0.36; ‘Δ%DL_CO_/yr’ *Rho* = 0.32). Moreover, ΔCRP/yr and ΔKL-6/yr showed a weak inverse or positive correlation with Δ6MWD/yr (‘ΔCRP/yr’ *Rho* = − 0.18; ‘ΔKL-6/yr’ *Rho* = 0.18). ΔSGRQ total/yr, ΔFVC (% predicted)/yr, ΔFEV_1_ (% predicted)/yr, and ΔDL_CO_ (% predicted)/yr were also correlated with ΔFBS/yr. At the baseline, the 6MWD was significantly and inversely associated with all domains of the SGRQ score and positively associated with FVC (% predicted), FEV_1_ (% predicted), and DL_CO_ (% predicted). Meanwhile, FBS was positively associated with all domains of the SGRQ score and CRP as well as inversely associated with DL_CO_ (% predicted). Incidentally, a slight negative correlation between Δ6MWD/yr and ΔFBS/yr (*Rho* = − 0.15) was observed; however, no apparent correlation between baseline 6MWD and FBS (*Rho* = − 0.05) existed.

**Table 2:** Longitudinal correlation between 6MWT parameters and anchors

ΔAnchor/yr	Δ6MWD/yr (m/yr)		ΔFBS/yr
(Longitudinal)	*Rho* (95% CI)		*Rho* (95% CI)
ΔSGRQ symptom/yr^*^	−0.07 (-0.21 to 0.07)		0.19 (0.05 to 0.32)
ΔSGRQ activity/yr^*^	−0.30 (-0.43 to -0.17)		0.10 (-0.04 to 0.24)
ΔSGRQ impact/yr^*^	−0.11 (-0.25 to 0.03)		0.14 (0 to 0.27)
ΔSGRQ total/yr^*^	−0.22 (-0.35 to -0.08)		0.21 (0.07 to 0.34)
Δ%FVC/yr^†^	0.29 (0.15 to 0.42)		−0.41 (-0.53 to -0.29)
Δ%FEV_1_/yr^†^	0.36 (0.22 to 0.48)		−0.38 (-0.50 to -0.25)
Δ%DL_CO_/yr^‡^	0.32 (0.18 to 0.44)		−0.23 (-0.36 to -0.09)
ΔCRP/yr^§^	−0.18 (-0.32 to -0.04)		0.09 (-0.06 to 0.23)
ΔKL-6/yr^||^	0.18 (0.04 to 0.32)		0.13 (-0.01 to 0.27)
Δ6MWD/yr	-		-0.15 (-0.28 to 0)

6MWT, six-minute walk test; SGRQ, St. George’s Respiratory Questionnaire; FVC, forced vital capacity; FEV_1_, forced expiratory volume in 1s; DL_CO_, diffusing capacity of the lung for carbon monoxide; CRP, serum C-reactive protein; KL-6, sialylated carbohydrate antigen Krebs von den Lungen-6; 6MWD, 6-minute walk distance

### Predicted changes in the 6MWD by anchors or variables at baseline

Table 3 shows the results from the linear mixed-effects models that extend the results from the association analyses by representing predicted 6MWD change in association with the anchors and the variables at baseline. Increases at baseline in all the SGRQ scores (worse HRQL) were predicted to decrease 6MWD. Conversely, for PFT, increases at baseline in %FVC and %DL_CO_ were predicted to increase 6MWD. Likewise, an increase at baseline in KL-6 was predicted to decrease 6MWD. In the variables at baseline, older age and currently undergoing treatment were the predictors for deterioration in the 6MWD. Considering the changes in FBS, increases at baseline in all the SGRQ scores (worse HRQL), CRP, and old age were predicted to yield aggravated FBS. Conversely, increases at baseline in %FVC and %DL_CO_ were predicted to improve FBS.

**Table 3:** Linear mixed-effects models-generated parameter estimates for the changes in the 6MWT parameters resulting from anchor score or variables at baseline

Anchors and Variables	Δ6MWD			ΔFBS	
	Estimate (95% CI)	*P*-value		Estimate (95% CI)	*P*-value
Anchors					
SGRQ symptom	−0.52 (− 0.83 to − 0.21)	0.001		0.01 (0.00 to 0.02)	0.001
SGRQ activity	−0.50 (− 0.82 to − 0.17)	0.003		0.02 (0.02 to 0.03)	< 0.001
SGRQ impact	−0.81 (− 1.20 to − 0.41)	< 0.001		0.02 (0.01 to 0.02)	< 0.001
SGRQ total	−0.83 (− 1.23 to − 0.43)	< 0.001		0.02 (0.01 to 0.03)	< 0.001
FVC (% predicted)	0.75 (0.33 to 1.16)	< 0.001		−0.01 (− 0.02 to 0.00)	0.016
FEV_1_ (% predicted)	0.39 (− 0.02 to 0.80)	0.065		−0.01 (− 0.02 to 0.00)	0.059
DL_CO_ (% predicted)	1.36 (0.87 to 1.85)	< 0.001		−0.03 (− 0.04 to − 0.02)	< 0.001
CRP (mg/dl)	−2.94 (− 14.04 to 8.16)	0.602		0.35 (0.14 to 0.56)	0.014
KL-6 (U/ml)	−0.05 (− 0.09 to − 0.02)	0.002		0.00 (0.00 to 0.00)	0.003
Variables					
Sex male (vs. female)	−14.35 (− 31.81 to 3.10)	0.059		0.51 (0.14 to 0.88)	0.595
Age	−2.37 (− 3.09 to − 1.65)	< 0.001		0.03 (0.02 to 0.05)	< 0.001
Disease duration (years)	−0.15 (− 1.73 to 1.43)	0.850		0.01 (− 0.03 to 0.04)	0.718
BMI	1.08 (− 1.51 to 3.67)	0.411		−0.03 (− 0.09 to 0.02)	0.264
Cavitary lesions (vs. none)	−10.07 (− 25.74 to 5.60)	0.115		0.61 (0.29 to 0.93)	0.224
Smear positive (vs. negative)	0.45 (− 11.46 to 12.36)	0.866		0.63 (0.40 to 0.87)	0.053
Currently treated (vs. never/previously treated)	−9.60 (− 20.69 to 1.49)	0.014		0.55 (0.32 to 0.78)	0.182

6MWT, six-minute walk test; BMI, body mass index; CI, confidence interval; CRP, serum C-reactive protein; DL_CO_, diffusing capacity of the lung for carbon monoxide; FBS, Final Borg Scale; FEV_1_, forced expiratory volume in 1s; FVC, forced vital capacity; KL-6, sialylated carbohydrate antigen Krebs von den Lungen-6; SGRQ, St. George’s Respiratory Questionnaire; 6MWD, 6-minute walk distance.

Linear mixed-effects models were performed to test for the influence of variables and anchors on the change in 6MWT parameters and adjusted for baseline 6MWT parameters as a covariate (fixed effects = year, variables or anchors, interaction effects between year and variables or anchors; random effects = id, id × year).

### Comparison of Δ6MWD based on differences in ΔSGRQ/yr, ΔPFT/yr, ΔBT/yr

**Fig. 2:**
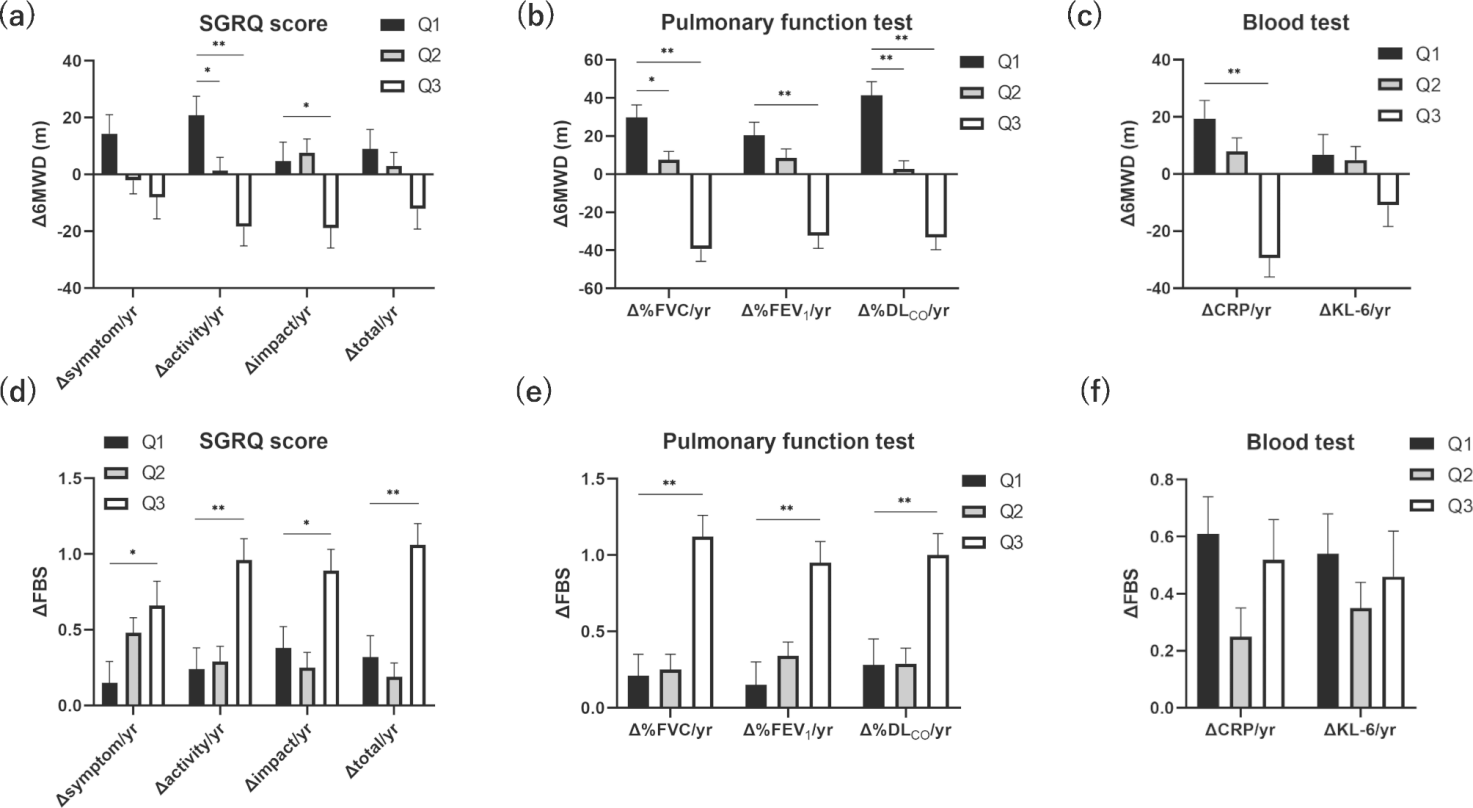
**(a-c)** Δ6MWD and **(d-f)** ΔFBS by quantiles of three different anchor change scores (Δanchor/yr) over observation period. **(a, d)** ΔSGRQ (symptom, activity, impact, and total)/yr, **(b, e)** pulmonary function test, **(c, f)** blood test. Δ, change score from baseline to final measurement during the observation period; Q1, Top 25% of patients with good change in anchor score over time (< 25th percentile); Q2, Middle 50% of patients with good change in anchor score over time (25th -75th percentile); Q3, Bottom 25% of patients with good change in anchor score over time (> 75th percentile); Alb, albumin; CRP, serum C-reactive protein; DL_CO_, diffusing capacity of the lung for carbon monoxide; FEV_1_, forced expiratory volume in 1s; FVC, forced vital capacity; KL-6, sialylated carbohydrate antigen Krebs von den Lungen-6; SGRQ, St. George’s Respiratory Questionnaire; yr, year; 6MWD, 6-minute walk distances. Δ6MWD estimate ± SE are calculated using mixed-effects model, adjusting for the baseline 6MWD as a covariate (fixed effects = year, groups [Q1 to Q3], interaction effects between year and groups; random effects = id, id × year). *P*-values represent one-way analysis of variance with post hoc comparisons using Tukey’s multiple comparison test. *P*-values were compared with quantile 1 (Q1). ^*^P < 0.05. ^**^P < 0.01

Figure 2 and Table S2 represent the mean changes in the 6MWD and FBS for subgroups stratified into three quantiles of changes in each anchor: the top 25% (Q1), middle 50% (Q2), and bottom 25% (Q3) groups. After adjusting for the baseline 6MWD, significant differences were observed in mean Δ6MWD based on quantiles of anchor change in ΔSGRQ activity/yr and ΔSGRQ impact/yr. For example, patients with a greater decline from baseline SGRQ activity scores (Q3) had significantly shorter 6MWD than those in Q1 (mean ± standard error [SE], − 18.44 ± 6.72, *P* < 0.001) (Fig. 2a). For FBS, there were significant ΔFBS differences between Q1 and Q3 in all ΔSGRQ/yr components (Fig. 2d).

**Fig. 3:**
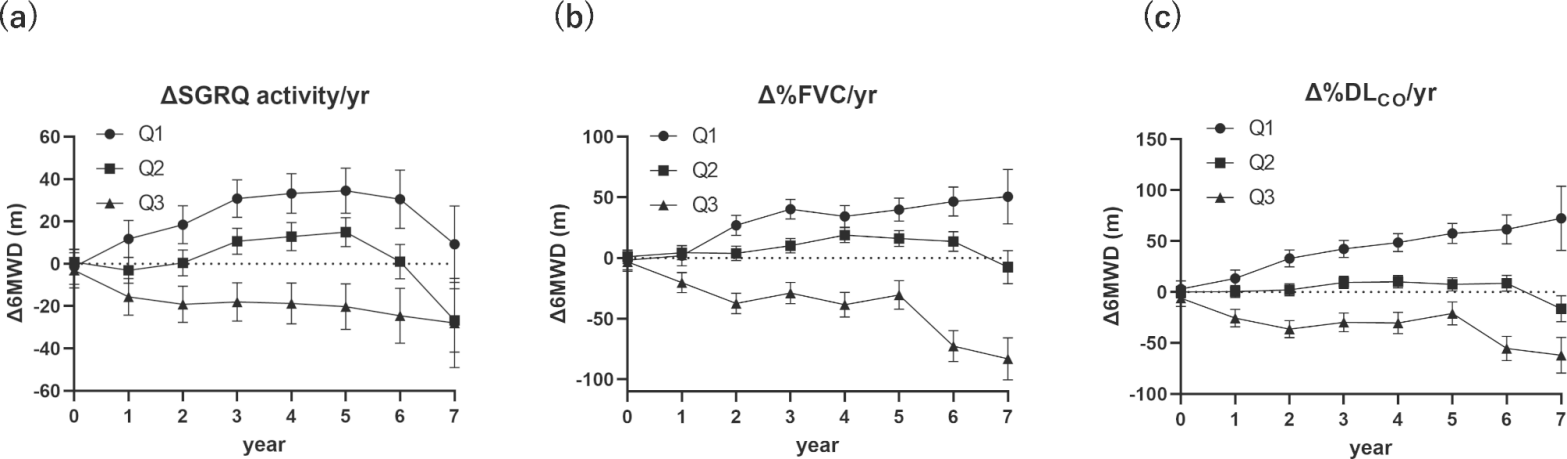
Change in 6MWD over time by quantiles of anchor change scores (Δanchor/yr). **(a)** ΔSGRQ activity/yr, **(b)** Δ%FVC/yr, (**c)** Δ%DL_CO_/yr. Δ, change score from baseline to each year; Q1, Top 25% of patients with good change in anchor score over time (< 25th percentile); Q2, Middle 50% of patients with good change in anchor score over time (25th -75th percentile); Q3, Bottom 25% of patients with good change in anchor score over time (> 75th percentile); DL_CO_, diffusing capacity of the lung for carbon monoxide; FVC, forced vital capacity; SGRQ, St. George’s Respiratory Questionnaire; yr, year; 6MWD, 6-minute walk distances. Δ6MWD estimate ± SE are calculated using mixed-effects model, adjusting for the baseline 6MWD as a covariate (fixed effects = year, groups [Q1 to Q3], interaction effects between year and groups; random effects = id, id × year). *P*-values represent one-way analysis of variance with post hoc comparisons using Tukey’s multiple comparison test

PFT changes over time (ΔPFT/yr) had significant differences in both Δ6MWD and ΔFBS between Q1 and Q3 for three measurement items (%FVC, %FEV_1_, and %DL_CO_) (Fig. 2b and e). ΔBT/yr had a significant difference in Δ6MWD for stratification based on ΔCRP/yr but not for ΔFBS, whereas for stratification based on ΔKL-6/yr, neither Δ6MWD nor ΔFBS had any difference (Fig. 2c and f). Figure 3 shows the longitudinal change in Δ6MWD of a representative anchor that showed significant changes. The difference from Q1 to Q3 tended to become more prominent over time.

## Discussion

In the present study, we collected up to 7 years of data from 188 patients with NTM-PD on 6MWT parameters (6MWD and FBS), clinical parameters including HRQL (SGRQ), PFT, and BT findings and performed longitudinal analyses. No large studies have been conducted on patients with NTM-PD characterized by routine and repeated evaluation of 6MWT under the same conditions. We elucidated that the 6MWT parameters over time are related to HRQL scores and PFT results. Noteworthily, even without special medical equipment or tools, repeated 6MWT measurements were observed to be conducive to understanding the patient profile.

Attempts have been made to correlate the 6MWT parameters with other clinical indicators in groups of patients with various cardiopulmonary diseases [31]. Because NTM-PD in particular is a process that often deteriorates slowly over a long period of time, some studies have been conducted to examine changes in exercise tolerance over time. Shah et al. reported that longitudinal change in EQ-5D-3 L, a comprehensive and generic measure of HRQL, did not correlate with longitudinal change in 6MWD in patients with NTM-PD [32]. However, we previously reported that the SGRQ scores have longitudinal validity in assessing disease severity and were sensitive to changes in patients with NTM-PD, particularly changes in %FEV_1_ [9]. That report, however, focused on changes in the SGRQ over time and did not incorporate 6MWT parameters in its analysis. We have also previously reported that the 6MWD and FBS scores are useful parameters for evaluating SGRQ scores in patients with NTM-PD [16], but we did not evaluate the prognosis of exercise capacity due to a lack of perspective of change over time. Thus, the significance of changes in 6MWD and FBS over time had not been clarified previously.

The most notable finding of this study was that when stratified into three quantiles of changes in each anchor (Q1, Q2, and Q3), 6MWT parameters worsened over time in the bottom 25% group (Q3). Here, the difference between Q1 and Q3 estimates of Δ6MWD for ΔSGRQ activity/yr, ΔPFT/yr, and ΔCRP/yr exceeded 35 m. This is greater than the minimum clinically important difference (MCID) of 6MWD for idiopathic pulmonary fibrosis [33], coronary artery disease [34], asthma [35], chronic heart failure [36], and chronic obstructive pulmonary disease (COPD) [37], making these parameters statistically and clinically relevant longitudinal changes. Alternatively, the MCID of the Borg scale is 1 unit for COPD [38]. In the present study, ΔFBS was worse in the bottom 25% group (Q3) of all ΔSGRQ/yr and all ΔPFT/yr. Especially in Q3 of ΔSGRQ total/yr, Δ%FVC/yr, and Δ%DL_CO_/yr, ΔFBS reached more than 1 unit, indicating exacerbation of dyspnea during exercise. Correlation analysis also supported this observation and showed robustness. In our study, only ΔSGRQ total/yr correlated with both Δ6MWD/yr and ΔFBS/yr in the longitudinal analyses. ΔSGRQ activity/yr had the strongest negative correlation with Δ6MWD/yr, while ΔSGRQ symptoms/yr had the opposite trend, correlating only with ΔFBS/yr. These differences are understandable based on the meaning of each SGRQ score domain. Anchor scores and variables at baseline that worsened Δ6MWD were higher SGRQ scores, lower %FVC, lower %DL_CO_, higher KL-6, older age, and undergoing treatment at registration. Similarly, these clinical parameters and elevated CRP, excluding undergoing treatment at registration, worsened ΔFBS. Ono et al. have reported that low pulmonary function and fibrocavitary type are associated with lower incremental shuttle walk test distance in patients with NTM-PD [39].

It is important that these results were obtained based on a study design adapted to the disease specificity of NTM, with a chronic clinical course and long-term follow-up requirement. This study provides physicians with novel information regarding the prognosis of exercise tolerance in patients with NTM-PD, which has been poorly characterized so far. Moreover, the associations characterized in this study should be disseminated to support physicians’ decisions in clinical practice. Relying solely on changes over time in SGRQ scores and PFT as clinical indicators is problematic. Owing to the intrinsic subjectivity of the SGRQ score, it is difficult to establish a universal method of interpreting change over time that can be adapted to all patients. In addition, in patients with advanced interstitial lung disease, the presence of the “floor effect” could impede the accurate reflection of subsequent changes in FVC within measurement results [40]. Particularly, in the area of drug discovery, a report from the United States Food and Drug Administration recommends that clinical trials focus more on improving daily functioning, such as 6MWT, as an outcome measure [41]. 6MWD is already the most commonly used primary endpoint in pulmonary hypertension [42], and some have suggested that this trend should be extended to other pulmonary diseases [40]. Reportedly, perioperative and post-discharge respiratory rehabilitation following surgical pneumonectomy for NTM-PD significantly improves 6MWD at 6 months postoperatively [43]. Hence, a proper understanding of the factors that alter 6MWT parameters in patients with NTM-PD may lead to further elucidation of respiratory physiology, effective rehabilitation, and development of novel therapies.

This study has several limitations. First, this study is a retrospective analysis of a limited number of cases from a single center and the time period included in the study is only part of the long disease course of NTM-PD. Second, it is possible that patients with more severe NTM-PD were excluded from the study, as we included patients who were able to undergo multiple 6MWTs. Third, the impact of introducing home oxygen therapy and respiratory rehabilitation was not considered. These interventions have been reported to improve 6MWD in patients with COPD and heart failure; however, there are no studies on their application in NTM-PD management [44, 45]. Future studies with data accumulated over a decade or more in larger patient populations would be desirable.

## Conclusions

The present study suggests that decreased walking distance and exacerbation of dyspnea on exertion over time in patients with NTM-PD may reflect a deterioration of HRQL and pulmonary function, affecting their ADLs. Consistently conducting the 6MWT to assess changes over time contributes to the physician’s understanding of the patient’s clinical profile.

## Electronic supplementary material

Below is the link to the electronic supplementary material.

Supplementary Material 1

## Data Availability

The datasets used and analyzed during the current study are available from the corresponding author on reasonable request.

## List of abbreviations

ADL: Activities of daily living
ALB: Albumin
ATS/ISDA: American Thoracic Society/Infectious Disease Society of America statements
BMI: Body mass index
BT: Blood test
COPD: Chronic obstructive pulmonary disease
CRP: Serum C-reactive protein
DL_CO_: Diffusing capacity for carbon monoxide
FBS: Final Borg Scale
FC: Fibrocavitary
FEV_1_: Forced expiratory volume in 1 s
FVC: Forced vital capacity
HR: Heart rate
HRCT: High-resolution computed tomography
HRQL: Health-related quality of life
IQR: Interquartile range
KL-6: Sialylated carbohydrate antigen Krebs von den Lungen-6
MAC: *Mycobacterium avium* complex
MCID: Minimum clinically important difference
NB: Nodular/bronchiectatic
NTM-PD: Nontuberculous mycobacterial pulmonary disease
PFT: Pulmonary function test
SGRQ: St. George’s Respiratory Questionnaire
SpO_2_: Oxygen saturation by pulse oximetry
6MWD: Six-minute walk distance
6MWT: Six-minute walk test
