# Supplementary material for: Longitudinal significance of six-minute walk test in patients with nontuberculous mycobacterial pulmonary disease: an observational study
Source: BMC Pulm Med. 2023 Jul 6;23:247. doi: 10.1186/s12890-023-02528-y (PMC10327387; doi:10.1186/s12890-023-02528-y)
Supplement: Supplementary file 1 — Supplementary Material 1 [file 12890_2023_2528_MOESM1_ESM.docx]

## **Table S1**

Cross-sectional correlation between the 6MWT parameters and anchors

| Anchor | 6MWD (m) |  | FBS |
| --- | --- | --- | --- |
| (Cross-sectional) | *Rho* (95% CI) |  | *Rho* (95% CI) |
| SGRQ symptom^*^ | −0.17 (-0.30 to -0.03) |  | 0.23 (0.09 to 0.37) |
| SGRQ activity^*^ | −0.35 (-0.47 to -0.22) |  | 0.31 (0.17 to 0.43) |
| SGRQ impact^*^ | −0.24 (-0.37 to -0.10) |  | 0.36 (0.23 to 0.48) |
| SGRQ total^*^ | −0.30 (-0.42 to -0.16) |  | 0.36 (0.23 to 0.48) |
| %FVC^†^ | 0.26 (0.13 to 0.39) |  | -0.12 (-0.26 to 0.02) |
| %FEV_1_^†^ | 0.23 (0.09 to 0.36) |  | -0.03 (-0.18 to 0.11) |
| % DL_CO_^‡^ | 0.36 (0.23 to 0.48) |  | -0.22 (-0.36 to -0.08) |
| CRP (mg/dl)^§^ | −0.11 (-0.25 to 0.04) |  | 0.18 (0.04 to 0.32) |
| KL-6 (U/ml)^\|\|^ | −0.09 (-0.23 to 0.06) |  | 0.14 (0 to 0.28) |
| 6MWD (m) | - |  | -0.05 (-0.19 to 0.09) |

CRP, serum C-reactive protein; DL_CO_, diffusing capacity of the lung for carbon monoxide; FBS, Final Borg Scale; FEV_1_, forced expiratory volume in 1s; FVC, forced vital capacity; KL-6, sialylated carbohydrate antigen Krebs von den Lungen-6; SGRQ, St. George’s Respiratory Questionnaire; yr, year; 6MWD, 6-minute walk distance.

Values are expressed as Spearman’s correlation coefficients (*Rho* and 95%CI).

^*^ n = 188; ^†^n = 185; ^‡^n = 183; ^§^n = 186; ^||^n = 184.

# **Table S2**

Mean changes in the 6MWT parameters by stratifying into 3 groups based on annual change in anchor scores

| Variable | n | Δ6MWD (m) |  |  |  |  | ΔFBS |  |  |  |
| --- | --- | --- | --- | --- | --- | --- | --- | --- | --- | --- |
|  |  | Estimate ± SE | P-value  (group, multiple comparison) | P-value  (group) | P-value  (years*group) |  | Estimate ± SE | P-value  (group, multiple comparison) | P-value  (group) | P-value  (years*group) |
| SGRQ symptom annual change |  |  |  | 0.054 | 0.202 |  |  |  | 0.037 | 0.098 |
| Q1 (≤-3.65) | 47 | 14.34 ± 6.66 | (Reference) |  |  |  | 0.15 ± 0.14 | (Reference) |  |  |
| Q2 (-3.65 to 6.56) | 94 | -2.08 ± 4.73 | 0.111 |  |  |  | 0.48 ± 0.10 | 0.114 |  |  |
| Q3 (>6.56) | 47 | -8.11 ± 7.53 | 0.069 |  |  |  | 0.66 ± 0.16 | 0.040 |  |  |
| SGRQ activity annual change |  |  |  | <0.001 | 0.046 |  |  |  | <0.001 | 0.387 |
| Q1 (≤-3.01) | 47 | 20.86 ± 6.65 | (Reference) |  |  |  | 0.24 ± 0.14 | (Reference) |  |  |
| Q2 (-3.01 to 5.93) | 93 | 1.35 ± 4.71 | 0.046 |  |  |  | 0.29 ± 0.10 | 0.953 |  |  |
| Q3 (>5.93) | 48 | -18.44 ± 6.72 | <0.001 |  |  |  | 0.96 ± 0.14 | <0.001 |  |  |
| SGRQ impact annual change |  |  |  | 0.007 | 0.093 |  |  |  | 0.001 | 0.738 |
| Q1 (≤-0.98) | 47 | 4.67 ± 6.70 | (Reference) |  |  |  | 0.38 ± 0.14 | (Reference) |  |  |
| Q2 (-0.98 to 4.72) | 94 | 7.70 ± 4.80 | 0.929 |  |  |  | 0.25 ± 0.10 | 0.701 |  |  |
| Q3 (>4.72) | 47 | -18.93 ± 7.00 | 0.041 |  |  |  | 0.89 ± 0.14 | 0.028 |  |  |
| SGRQ total annual change |  |  |  | 0.084 | 0.183 |  |  |  | <0.001 | 0.275 |
| Q1 (≤-1.08) | 47 | 8.94 ± 6.89 | (Reference) |  |  |  | 0.32 ± 0.14 | (Reference) |  |  |
| Q2 (-1.08 to 4.38) | 94 | 3.00 ± 4.76 | 0.758 |  |  |  | 0.19 ± 0.09 | 0.706 |  |  |
| Q3 (>4.38) | 47 | -12.20 ± 7.02 | 0.083 |  |  |  | 1.06 ± 0.14 | <0.001 |  |  |
| FVC (% predicted) annual change |  |  |  | <0.001 | <0.001 |  |  |  | <0.001 | <0.001 |
| Q1 (≥ 0.57%) | 46 | 29.84 ± 6.47 | (Reference) |  |  |  | 0.21 ± 0.14 | (Reference) |  |  |
| Q2 (-1.56% to 0.57%) | 93 | 7.54 ± 4.41 | 0.013 |  |  |  | 0.25 ± 0.10 | 0.968 |  |  |
| Q3 (< -1.56%) | 46 | -39.31 ± 6.41 | <0.001 |  |  |  | 1.12 ± 0.14 | <0.001 |  |  |
| FEV_1_ (% predicted) annual change |  |  |  | <0.001 | <0.001 |  |  |  | <0.001 | 0.002 |
| Q1 (≥ 0.50%) | 46 | 20.41 ± 6.85 | (Reference) |  |  |  | 0.15 ± 0.15 | (Reference) |  |  |
| Q2 (-1.45% to 0.50%) | 93 | 8.67 ± 4.54 | 0.328 |  |  |  | 0.34 ± 0.09 | 0.527 |  |  |
| Q3 (< -1.45%) | 46 | -32.30 ± 6.67 | <0.001 |  |  |  | 0.95 ± 0.14 | <0.001 |  |  |
| DL_CO_ (% predicted) annual change |  |  |  | <0.001 | <0.001 |  |  |  | <0.001 | 0.004 |
| Q1 (≥ 3.91%) | 46 | 41.47 ± 7.07 | (Reference) |  |  |  | 0.28 ± 0.17 | (Reference) |  |  |
| Q2 (0% to 3.91%) | 92 | 2.75 ± 4.31 | <0.001 |  |  |  | 0.29 ± 0.10 | 0.998 |  |  |
| Q3 (< 0%) | 45 | -33.26 ± 6.36 | <0.001 |  |  |  | 1.00 ± 0.14 | 0.004 |  |  |
| CRP annual change |  |  |  | <0.001 | <0.001 |  |  |  | 0.073 |  |
| Q1 (≤ 0 mg/dl) | 50 | 19.44 ±6.35 | (Reference) |  |  |  | 0.61 ± 0.13 | (Reference) |  |  |
| Q2 (0 mg/dl to 0.04 mg/dl) | 90 | 7.97 ±4.64 | 0.314 |  |  |  | 0.25 ± 0.10 | 0.086 |  |  |
| Q3 (> 0.04 mg/dl) | 46 | -29.52 ± 6.58 | <0.001 |  |  |  | 0.52 ± 0.14 | 0.886 |  |  |
| KL-6 annual change |  |  |  | 0.145 | 0.849 |  |  |  | 0.523 |  |
| Q1 (≤ -3.40 U/ml) | 45 | 6.79 ± 7.02 | (Reference) |  |  |  | 0.54 ± 0.14 | (Reference) |  |  |
| Q2 (-3.40 U/ml to 16.58 U/ml) | 93 | 4.92 ± 4.72 | 0.973 |  |  |  | 0.35 ± 0.09 | 0.517 |  |  |
| Q3 (> 16.58 U/ml) | 46 | -10.95 ± 7.43 | 0.194 |  |  |  | 0.46 ± 0.16 | 0.934 |  |  |
| Δ, change score from baseline to final measurement during the observation period; CRP, serum C-reactive protein; DL_CO_, diffusing capacity of the lung for carbon monoxide; FBS, Final Borg Scale; FEV_1_, forced expiratory volume in 1s; FVC, forced vital capacity; KL-6, sialylated carbohydrate antigen Krebs von den Lungen-6; SE, standard error; SGRQ, St. George’s Respiratory Questionnaire; 6MWD, 6-minute walk distance.  Estimates for the changes in the 6MWT parameters ± SE are calculated using mixed-effects model, adjusting for the baseline 6MWT parameters as a covariate (fixed effects=year, group, year*group; random effects=id, id*year). P-values represent one-way analysis of variance with post hoc comparisons using Tukey's multiple comparison test. Q1: <25^th^ percentile, Q2: 25^th^-75^th^ percentile, Q3: >75^th^ percentile. | | | | | | | | | | |
